# The 3' Untranslated Region of the Cyclin B mRNA Is Not Sufficient to Enhance the Synthesis of Cyclin B during a Mitotic Block in Human Cells

**DOI:** 10.1371/journal.pone.0074379

**Published:** 2013-09-13

**Authors:** Dominik Schnerch, Marie Follo, Julia Felthaus, Monika Engelhardt, Ralph Wäsch

**Affiliations:** Department of Hematology, Oncology and Stem Cell Transplantation, University Medical Center, Freiburg, Germany; Florida State University, United States of America

## Abstract

Antimitotic agents are frequently used to treat solid tumors and hematologic malignancies. However, one major limitation of antimitotic approaches is mitotic slippage, which is driven by slow degradation of cyclin B during a mitotic block. The extent to which cyclin B levels decline is proposed to be governed by an equilibrium between cyclin B synthesis and degradation. It was recently shown that the 3' untranslated region (UTR) of the murine cyclin B mRNA contributes to the synthesis of cyclin B during mitosis in murine cells. Using a novel live-cell imaging-based technique allowing us to study synthesis and degradation of cyclin B simultaneously at the single cell level, we tested here the role of the human cyclin B 3'UTR in regulating cyclin B synthesis during mitosis in human cells. We observed that the cyclin B 3'UTR was not sufficient to enhance cyclin B synthesis in human U2Os, HeLa or hTERT RPE-1 cells. A better understanding of how the equilibrium of cyclin B is regulated in mitosis may contribute to the development of improved therapeutic approaches to prevent mitotic slippage in cancer cells treated with antimitotic agents.

## Introduction

Mitotic exit is controlled by proteasomal degradation of the anaphase-promoting complex/cyclosome (APC/C) substrate proteins cyclin B and securin [1]. APC/C-dependent substrate degradation is blocked by the spindle assembly checkpoint (SAC) until every single chromosome has formed a stable bipolar attachment to the mitotic spindle [2-4]. While rapid degradation of cyclin B triggers mitotic exit once the SAC is satisfied, cyclin B degradation also takes place with slower kinetics in the presence of an active SAC [5-7]. Importantly, slow degradation of cyclin B was associated with an escape from the mitotic block and allows cells to enter G1 phase without chromosome segregation [5,7,8]. This escape mechanism, known as mitotic slippage, may give rise to the development of tetraploid cells and genomic instability [5,7-10].

Proteasomal degradation of cyclin B mediated by the APC/C is the major driving force in regulating mitotic exit [11,12]. In addition, several reports provided evidence that cyclin B is actively synthesized in mitosis both in human HeLa and U2Os cells as well as in murine cells [13-15]. Synthesis of cyclin B was shown to rely on the 3' untranslated region (UTR) of cyclin B mRNA in murine cells [14]. In agreement with this finding, cyclin B mRNA was shown to be subject to cytoplasmic polyadenylation in extracts from the human breast cancer cell line MCF-7 [16]. Cytoplasmic polyadenylation leads to poly(A) tail elongation which promotes translation and requires both a hexanucleotide sequence (AATAAA) and a cytoplasmic polyadenylation element (CPE; T_4-5_A_1-2_T) in the 3'UTR of the mRNA [17]. We and others could show that increased cyclin B levels contribute to a mitotic block [18,19]. These findings suggest that the strength of a mitotic block is governed by the cyclin B expression level, which represents an equilibrium between the synthesis and slow degradation of cyclin B [7,18]. Since translational control of inherited mRNAs by cytoplasmic polyadenylation is an evolutionarily conserved mechanism both in vertebrates and invertebrates [16,17,20], it is conceivable that 3'UTR-dependent translation of cyclin B during mitosis contributes to a mitotic block in human cells.

Antimitotic agents are frequently used to treat solid tumors and hematologic malignancy [10,21]. Mitotic slippage is known to be one major limitation of current antimitotic approaches contributing to treatment resistance, increased genetic instability and tumor progression [10,21]. In this study, we tested the role of the human cyclin B 3'UTR and its implications for cyclin B synthesis during a mitotic block in human U2Os, HeLa and hTERT RPE-1 cells using live-cell imaging at the single cell level to better understand how the equilibrium of cyclin B, which underlies mitotic slippage, is regulated.

## Material and Methods

### Generation of expression plasmids

The double-chimeric fusion proteins pLNCX2 Cyclin B mut5 YFP SNAP was established based on the pLNCX2 Cyclin B mut5 SNAP construct that has been described elsewhere [7]. To introduce the coding sequence for YFP into the Cyclin B reporter molecule, YFP was PCR-amplified using AAAAAAAAGCTTATGGTGAGCAAGGGCGAGGAG as a sense primer and AAAAAAAAGCTTCTTGTACAGCTCGTCCATGCC as a reverse primer. pMyrPalm-YFP (kindly provided by R. Tsien, HHMI UCSD, La Jolla) was used as a template. The PCR product was processed using a HindIII digest and placed in frame between the Cyclin B coding sequence and the sequence encoding the SNAP linker into the pLNCX2 Cyclin B mut5 SNAP construct. We established two different reporter constructs: Cyclin B YFP SNAP and cyclin B GFP SNAP. We tested the combination of BFP (histone marker), GFP (cyclin B expression), TMR-Star (SNAP substrate) and BG430 (SNAP substrate), YFP (cyclin B expression), mCherry (histone marker) and found the latter more red-shifted combination to cause less phototoxicity.

pMXs H2B mCherry IRES Blasticidin was established based on pH2B mCherry IRES neo3 (kindly provided by D. Gerlich, IMBA, Vienna). H2B mCherry was PCR-amplified using AAAAAAAGATCTGCCACCATGCCAGAGCCAGCGAAGTC as a sense primer and AAAAAACTCGAGTTACTTGTACAGCTCGTCCA as a reverse primer and pH2B mCherry IRES puro2 as a template. The PCR product was processed using a BglII/XhoI digest and introduced into the linearized pMXs IRES Blasticidin backbone (Cell Biolabs), which was linearized using a BamHI/XhoI digest.

To introduce the conserved region of the 3'UTR of human cyclin B into the construct adjacent to the coding sequence, the pLNCX2 Cyclin B mut5 YFP/GFP SNAP plasmid was linearized using a NotI/SalI digest. The 3'UTR sequence was generated by oligonucleotide annealing and sticky end overhangs at both ends of the annealed oligonucleotides facilitated ligation into the linearized backbone. The top strand oligonucleotide was ggccgccttgtaaacttgagttggagtactatatttacaaataaaattggcaccatgtgccatctgtg and the bottom strand oligonucleotide used was tcgacacagatggcacatggtgccaattttatttgtaaatatagtactccaactcaagtttacaaggc.

The complete cyclin B 3'UTR sequence was cloned from miRNA 3'UTR target clone HmiT021727-MT05 (GeneCopoeia Inc.) using TTTTTTGCGGCCGCCTTGTAAACTTGAGTTGGAG as a sense primer and TTTTTTGTCGACGTATTTGAGTATTGTTTTAT as a reverse primer. The PCR product was processed using a NotI/SalI digest and ligated into Cyclin B mut 5 YFP/GFP SNAP.

The conserved cyclin B 3'UTR sequence was as follows: cttgtaaacttgagttggagtactatatttacaaataaaattggcaccatgtgccatctgt.

The complete cyclin B 3'UTR sequence was as follows: cttgtaaacttgagttggagtactatatttacaaataaaattggcaccatgtgccatctgtacatattactgttgcatttacttttaataaagcttgtggccccttttacttttttatagcttaactaatttgaatgtggttacttcctactgtagggtagcggaaaagttgtcttaaaaggtatggtggggatatttttaaaaactccttttggtttacctggggatccaattgatgtatatgtttatatactgggttcttgttttatatacctggcttttactttattaatatgagttactgaaggtgatggaggtatttgaaaattttacttccataggacatactgcatgtaagccaagtcatggagaatctgctgcatagctctattttaaagtaaaagtctaccaccgaatccctagtccccctgttttctgtttcttcttgtgattgctgccataattctaagttatttacttttaccactatttaagttatcaactttagctagtatcttcaaactttcactttgaaaaatgagaattttatattctaagccagttttcattttggttttgtgttttggttaataaaacaatactcaaatac.

### Retroviral transduction

For generation of retrovirus-containing cell supernatant Phoenix-Ampho cells were transfected with calcium phosphate-precipitated plasmid DNA in the presence of 20µM chloroquine. Virus-containing supernatant was collected after 24 and 48 hours. For transduction U2Os, HeLa and hTERT RPE-1 cells were incubated in 4mL virus-containing supernatant supplemented with 2mL fresh normal growth medium and 5µg/mL hexadimethrine bromide.

### Cell culture and antibiotic selection

U2Os [7,22,23], HeLa (gift from M. Trepel, UKE Hamburg) and hTERT RPE-1 (gift from S. Linder, Karolinska Institute, Stockholm) cells were modified to express Histone H2B-mCherry and the double chimeric fusion protein Cyclin B mut5 YFP SNAP (later referred to as CYS cells) or CYS with the 3’ UTR sequence also added to the vector. U2Os cells were grown in complete DMEM medium (supplemented with 10% FCS, penicillin/streptomycin, sodium pyruvate, L-glutamine) in the presence of 10µg/mL blasticidin and 800µg/mL geneticin. HeLa cells were grown in complete DMEM medium in the presence of 6µg/mL blasticidin and 750µg/mL geneticin. hTERT RPE-1 cells were grown in complete DMEM:F12 medium in the presence of 7.5µg/mL blasticidin and 500µg/mL geneticin. Mitotic block release experiments were done as previously described [7]. To induce a mitotic block, nocodazole was applied at a final concentration of 200ng/mL and taxol was applied at a final concentration of 1µM.

### Western blotting

Western blot analyses were performed as previously described [23] using anti-cyclin B (Santa Cruz Biotechnology), anti-alpha-Tubulin (Sigma-Aldrich) as primary antibodies. Horseradish-peroxidase-conjugated secondary antibodies used were anti-rabbit (Amersham Biosciences), anti-mouse (Sigma-Aldrich). Densitometric quantification was performed using ImageJ.

### Live-cell imaging

Cells were seeded onto 8-well microscopy chambers (Ibidi, 80826 or 80822) at 25.000 or 50.000 per well and grown for 24 or 48 hours at standard cell culture conditions (37°C, 5% CO_2_, 100% air humidity) [7,22]. For BG 430 labeling cells were stained on 8 well microscopy chambers for 25 minutes in 200µL of prewarmed phenol red-free DMEM supplemented with 10% fetal bovine serum, penicillin/streptomycin, sodium pyruvate and 1µL BG 430 Stock Solution (1mM in DMSO) (New England Biolabs) resulting in a final labeling concentration of 5µM BG 430. Cells were washed four times in prewarmed phenol red-free DMEM supplemented with 10% fetal bovine serum, penicillin/streptomycin and sodium pyruvate with a final washing step of 30 minutes in DMEM^GFP^ (Evrogen) supplemented with 10% fetal bovine serum and L-glutamine. The medium was changed again (final imaging in DMEM^GFP^) to clear the cells from residual unbound SNAP substrate, which had diffused out of the cells. Image acquisition was performed on an Olympus IX-81 inverse microscope with climate chamber, using a UPLSAPO 20x objective (N.A. 0.75) and the Scan^R Acquisition software (v.2.2.09). The filter set used for monitoring of Histone H2B-mCherry had a 575/25 nm excitation filter and 600-662 nm emission filter. The filter set used for the analysis of BG 430 and YFP was the CFP/YFP dual bandpass filter set from Olympus. Image acquisition was repeated every 4 or 6 minutes under standard climate chamber conditions (37°C, 5% CO_2_, 60% air humidity). Read-outs were performed using the Scan^R Analysis software (v.1.2.0.6) allowing export of the time series from a desired cell and of the specific mean fluorescence intensity values.

### Data acquisition and statistics

Raw data of BG 430 fluorescence intensity were exported to Microsoft Excel 2002 for further calculations. For assessment of degradation slopes during a mitotic arrest, raw fluorescence intensity data of representative mitoses were exported and mean fluorescence intensity values were calculated. Raw data acquired from cells during a mitotic block were normalized based on maximum fluorescence intensity. Raw data acquired from cells undergoing unperturbed mitoses were normalized based on maximum fluorescence intensity and subject to background subtraction. Background levels for BG430 and YFP fluorescence intensity were estimated by calculation of mean nadir fluorescence intensity levels in G1 phase.

To exclude the possibility that differing amounts of photobleaching on the two signals had an unintended, hidden effect on our results, we looked at BG430 and YFP signals in the non-dividing cells over time. We noted no significant differences between the bleaching of BG430 and YFP.

## Results

### Generation of a double-chimeric cyclin B reporter allowing the study of cyclin B kinetics in the presence and absence of the endogenous 3' UTR

**Figure 1:**
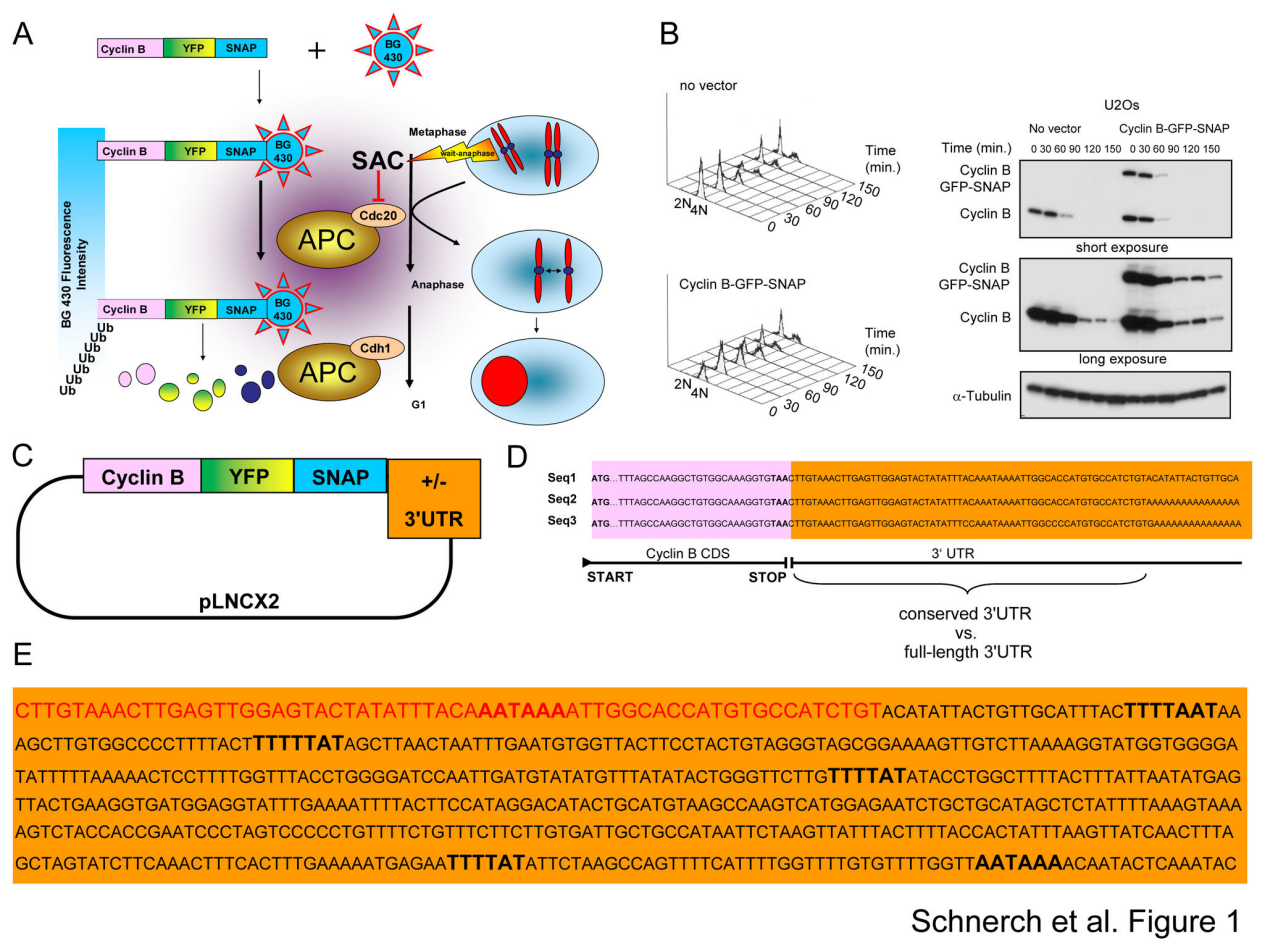
A double-chimeric system allowing the study of cyclin B kinetics at the single cell level in the presence and absence of the cyclin B 3'UTR. (A) A double-chimeric cyclin B reporter protein allowed the monitoring of whole protein expression by YFP fluorescence and degradation by pulse-chase labeling with the membrane-permeable SNAP substrate BG430. (B) Exemplary protein kinetics of the double-chimeric cyclin B reporter with GFP-SNAP were assessed by Western Blotting. Expression of the reporter protein and endogenous cyclin B following a nocodazole release are shown. (C) Plasmid map of the retroviral pLNCX2 vector indicating the sequence encoding the double-chimeric reporter and the 3' untranslated region (UTR). (D) Listing of different 3'UTRs found in available cyclin B full-length clones. (E) Sequence of the full-length 3'UTR indicating the sequence of the short 3'UTR variant in red and the hexanucleotide sequences (AATAAA) and the CPEs (T_4-5_A_1-2_T) in bold.

To address expression, synthesis and degradation of cyclin B simultaneously at the single cell level, we generated a SNAP tag-based double-chimeric reporter system, which is delivered by retroviral stable genomic integration (Figure 1A). The reporter construct encodes the endogenous cyclin B sequence as well as a YFP sequence, allowing the monitoring of cyclin B expression, and a sequence coding for a SNAP linker to allow pulse-chase labeling with fluorescent SNAP substrates to study degradation (Figure 1A).

In previous work we expressed knockdown-resistant SNAP-tagged cyclin B in combination with the knockdown of endogenous cyclin B to ensure cyclin B expression levels as close to wild type as possible to exclude any artificial effects, since protein overexpression has in general the potential to interfere with the phenotype. However, we observed in these experiments that C-terminal tagged cyclin B showed the same kinetic and mitotic timing with and without the additional knockdown of endogenous cyclin B [7]. Therefore, we chose in the current work a more straightforward approach without additional cyclin B knockdown. Since it is not feasible to manipulate cells with more than 2 or 3 retroviral vectors, the cells from the current study remain more amenable for further genetic manipulation.

To ensure that a double-chimeric cyclin B reporter reflects kinetics of wild-type cyclin B we performed Western Blotting following a release from a nocodazole block (Figure 1B and Figure S1). The cyclin B reporter molecules exhibited the same degradation kinetics as that seen for endogenous cyclin B, which was a prerequisite for our study (Figure 1B and Figure S1). The reporter protein will from now on be referred to as Cyclin B YFP SNAP (CYS). Since the 3’ UTR was shown to regulate cyclin B synthesis in different species in mitosis, we included the endogenous human cyclin B 3’ UTR sequence adjacent to the CYS coding sequence to compare the expression from these cassettes with that of the blank CYS expression cassette allowing us to delineate 3'UTR-specific effects (Figure 1C). The two 3’ UTRs were identified by screening the sequences of available *homo sapiens* full-length cyclin B clones (Figure 1D). This screen led to the identification of a long and a conserved, shorter 3’ UTR version. The shorter 3’ UTR was identical to the first 62 nucleotides of the long 3’ UTR version (Figure 1E; shorter 3'UTR in red, longer 3'UTR in black). While the shorter 3'UTR only harbored one hexanucleotide sequence, the longer UTR comprised two hexanucleotide sequences and four CPEs (Figure 1E) both of which are requirements for polyadenylation and enhanced translation.

### Degradation and synthesis of CYS throughout the cell cycle

**Figure 2:**
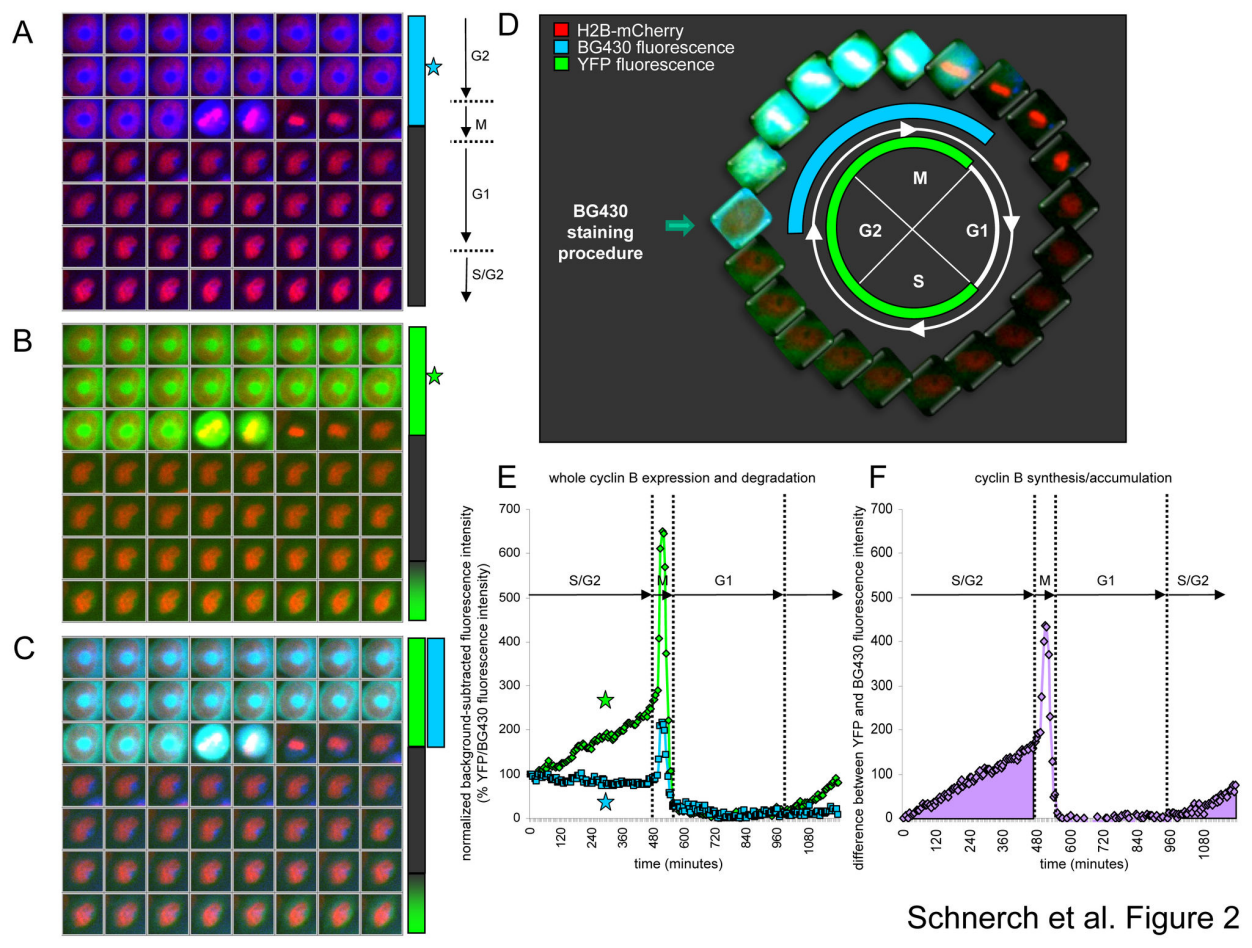
Degradation and synthesis of the double-chimeric cyclin B reporter throughout the cell cycle by live-cell imaging at the single-cell level. (A) Degradation of the reporter is reflected by a decrease in BG430 fluorescence intensity at the metaphase to anaphase transition following pulse-chase labeling. (B) Whole protein expression of the CYS reporter molecule is reflected by YFP fluorescence intensity throughout the cell cycle. (C) An overlay of YFP (green), BG430 (blue) and mCherry (red) fluorescence is depicted by the lower image series. (D) Whole protein expression, degradation and synthesis/accumulation of the CYS reporter and pulse-chase labeling with the SNAP substrate is depicted for the indicated cell cycle stages by overlaying of YFP and BG430 fluorescence. (E) YFP (green curve) and BG430 (blue curve) fluorescence intensity curves are shown and the corresponding cell cycle stages are indicated (see cell cycle stage between the dotted lines). Background fluorescence was subtracted and the initial fluorescence intensity level was set to 100%. (F) A curve displaying the synthesis rate/accumulation of the cyclin B reporter (as defined by the difference between YFP- and BG430-fluorescence) is indicated.

Labeling of CYS expressing reporter cells with the SNAP substrate BG430 results in (blue) cells entering mitosis (Figure 2A). During mitotic exit, the BG430 fluorescence intensity declined rapidly and was maintained at a low level until a slow increase in YFP-fluorescence intensity (green) indicated re-accumulation of CYS later during the subsequent cell cycle (Figure 2B and Movie S1). The overlay is shown in Figure 2C. A schematic of the procedure is shown in Figure 2D. While BG430- and YFP-fluorescence dropped in a comparable manner during mitotic exit, BG430- and YFP-fluorescence intensity curves separated during pre-mitotic accumulation (Figure 2E). Since we were able to measure both BG430- and YFP-fluorescence intensities, we could estimate the accumulation of CYS protein by calculating the difference between whole-protein CYS expression (YFP-fluorescence) and CYS degradation (BG430-fluorescence intensity) (Figure 2F). YFP- and BG430-fluorescence intensity curves were normalized based on the initial fluorescence intensity (Figure 2F). The CYS accumulation could be determined within a window of isovolumetry during interphase and mitosis but not during mitotic entry and exit when the cell unterwent changes in its shape [7]. The mean YFP- and BG430-fluorescence intensities were acquired from identical objects at the same time, leading to high accuracy for our synthesis rates. This allowed us to detect even subtle synthesis activity.

### Cyclin B slow degradation occurs during chromosomal misalignment

**Figure 3:**
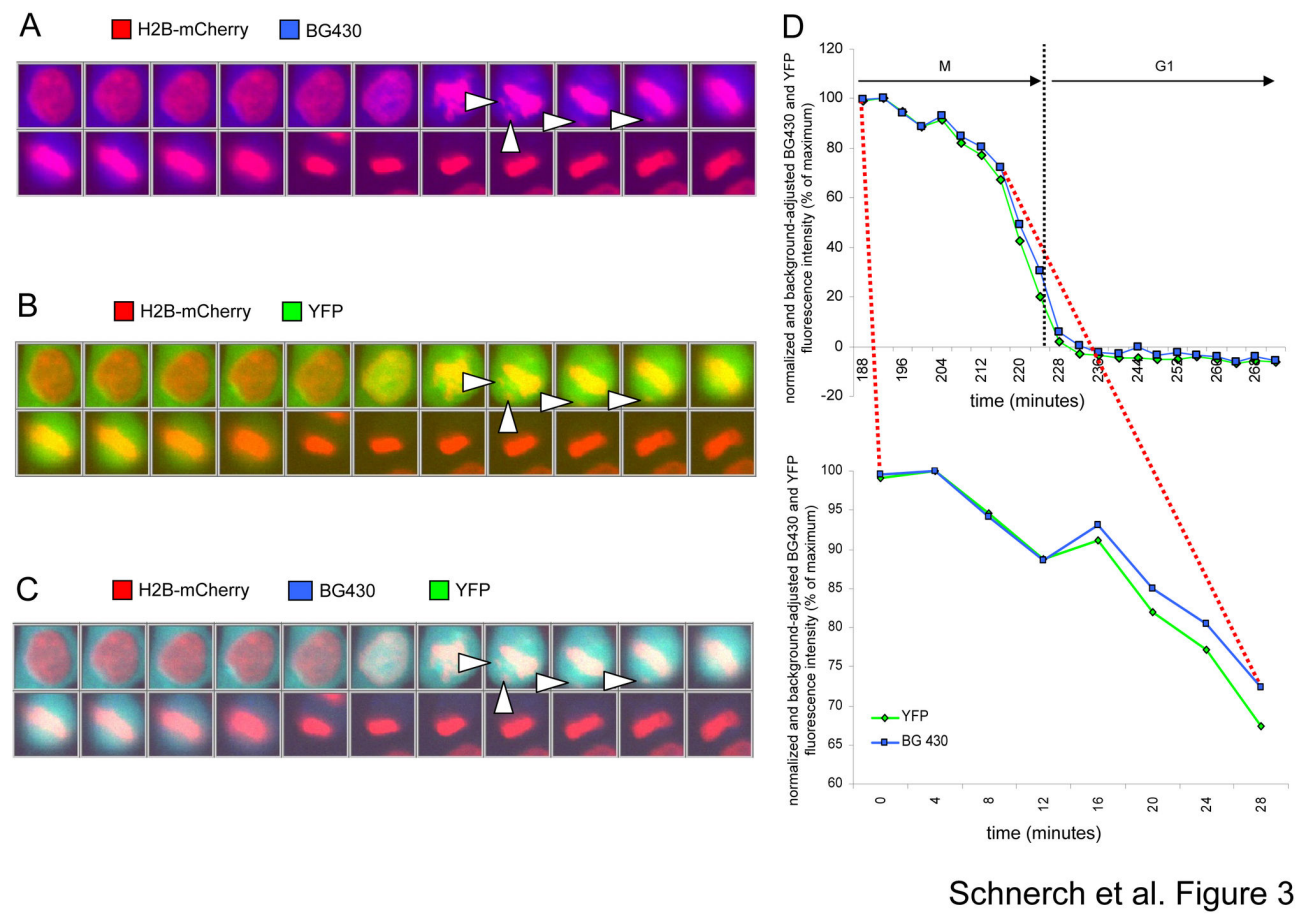
Monitoring whole protein expression and degradation of the CYS reporter in the presence of spontaneous chromosomal misalignment in mitosis in U2Os cells. (A) Degradation of the reporter is reflected by a decrease in BG430 fluorescence intensity in a single cell showing misalignment of single chromosomes (white arrowheads, chromosomes appear in red). (B) Whole protein expression of the cyclin B reporter molecule is reflected by YFP fluorescence intensity. (C) Overlay of YFP and BG430 fluorescence. (D) YFP and BG430 fluorescence intensity curves for the cell depicted in A, B and C during mitosis (upper diagram). Fluorescence intensities during the metaphase delay are indicated as an enlargement in the lower diagram (enlarged section of the upper curve is indicated by the red dotted line).

We recently reported that slow degradation of cyclin B is associated with chromosomal misalignment during metaphase in U2Os cells [7]. Here, we depict a representative cell showing misalignment of single chromosomes in metaphase (Figure 3A, white arrowheads indicate chromosomal misalignments). During metaphase delay, we observed that both BG430-fluorescence intensity, a decrease of which reflects CYS degradation (Figure 3A), as well as YFP-fluorescence intensity, indicating CYS whole protein expression (Figure 3B), were kept at high levels. Once every single chromosome had attached to the mitotic spindle fluorescence intensities dropped in a prompt manner (Figure 3A, B, C). Fluorescence intensity curves behaved in a very similar fashion during prometaphase/metaphase. The depicted cell expressed CYS from an expression cassette lacking the human endogenous cyclin B 3'UTR sequence (Figure 3D). Background fluorescence, as defined by the mean fluorescence level in G1 phase, was subtracted and intensity levels were normalized based on maximum fluorescence intensity in mitosis.

### The human endogenous cyclin B 3'UTR sequence is not sufficient to mediate synthesis of cyclin B in the presence of a mitotic block in U2Os cells

**Figure 4:**
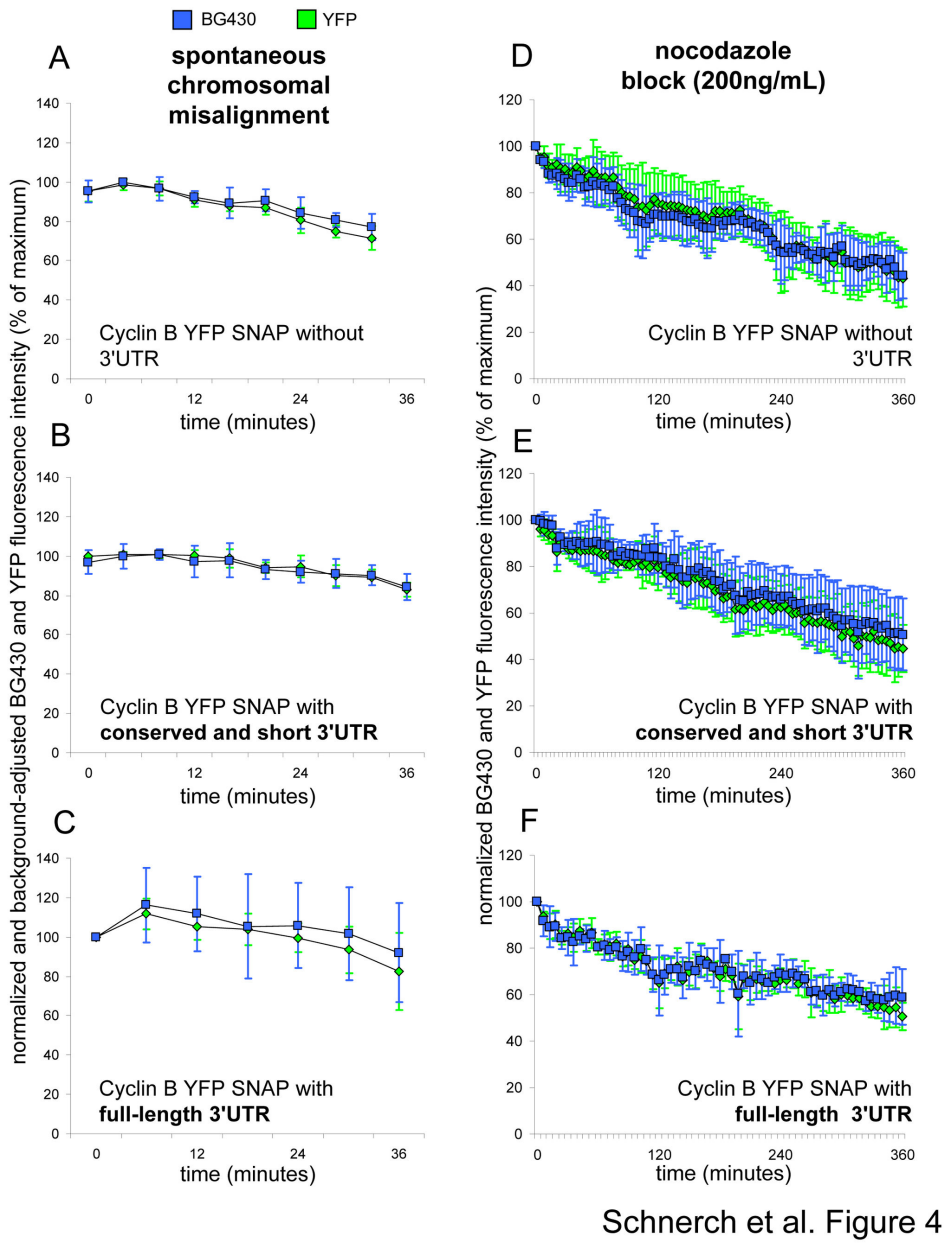
Whole protein expression and degradation of the cyclin B reporter during a mitotic delay/block in the presence or absence of the cyclin B 3'UTR in U2Os cells. YFP and BG430 fluorescence intensity curves as determined by live-cell imaging. (A, D) The investigated cells express the CYS reporter without an endogenous cyclin B 3'UTR, (B, E) with a short/conserved (C, F) and a long 3'UTR sequence. Cells displaying spontaneous chromosomal misalignment that indicates an active SAC are shown in A-C (mean fluorescence intensities +/- SD are indicated, n=4). Cells that are arrested in a mitotic block using a final nocodazole concentration in the growth medium of 200ng/mL are depicted in D-F (mean fluorescence intensities +/- SD are indicated, n=4). For every shown experimental setting the analyzed cells are representatives of three independent experiments each of which included at least 50.000 cells.

In order to study the effect of the human endogenous cyclin B 3'UTR on the synthesis of CYS during a mitotic block, we established retroviral plasmids encoding conserved short or long variants of the human cyclin B 3'UTR. The 3'UTR sequences were ligated into the expression plasmid adjacent to the 3' terminus of the CYS coding sequence (Figure 1C and Materials and Methods section) to ensure that the 3'UTR sequences are encoded by the mRNAs, which are transcribed from the expression cassette. First, we addressed both whole CYS expression (YFP-fluorescence) and degradation kinetics (BG430-fluorescence) in cells harboring an expression cassette lacking a human 3'UTR sequence (Figure 4A and 4D). We studied YFP- and BG430-fluorescence during CYS slow degradation in unperturbed cells exhibiting spontaneous chromosomal misalignment (Figure 4A). To establish a setting where cells are kept in a mitotic block for a longer time cells were exposed to nocodazole, which induced a mitotic block by spindle disruption (Figure 4D). Cells exhibiting a mitotic delay due to chromosomal misalignment did not reveal a difference in cyclin B kinetics as assessed by YFP- and BG430-fluorescence intensity (Figure 4A). Likewise, we did not note a difference between YFP- and BG430-fluorescence intensities in nocodazole-treated cells (Figure 4D). To our surprise in cells expressing the CYS reporter protein from an expression cassette encoding the conserved shorter 3'UTR variant, we did not detect differences in kinetics as assessed by YFP- and BG430-fluorescence intensities (Figure 4B and 4E). Moreover, we did not observe differences between YFP- and BG430-fluorescence intensities even in cells harboring an expression cassette encoding the long 3'UTR sequence (Figure 4C and 4F). This indicates that both tested 3'UTR variants were unable to induce synthesis of the CYS reporter protein in U2Os cells during a mitotic block.

### Insufficiency of the human endogenous cyclin B 3'UTR sequence to mediate synthesis of cyclin B in a mitotic block in human HeLa and hTERT RPE-1 cells

**Figure 5:**
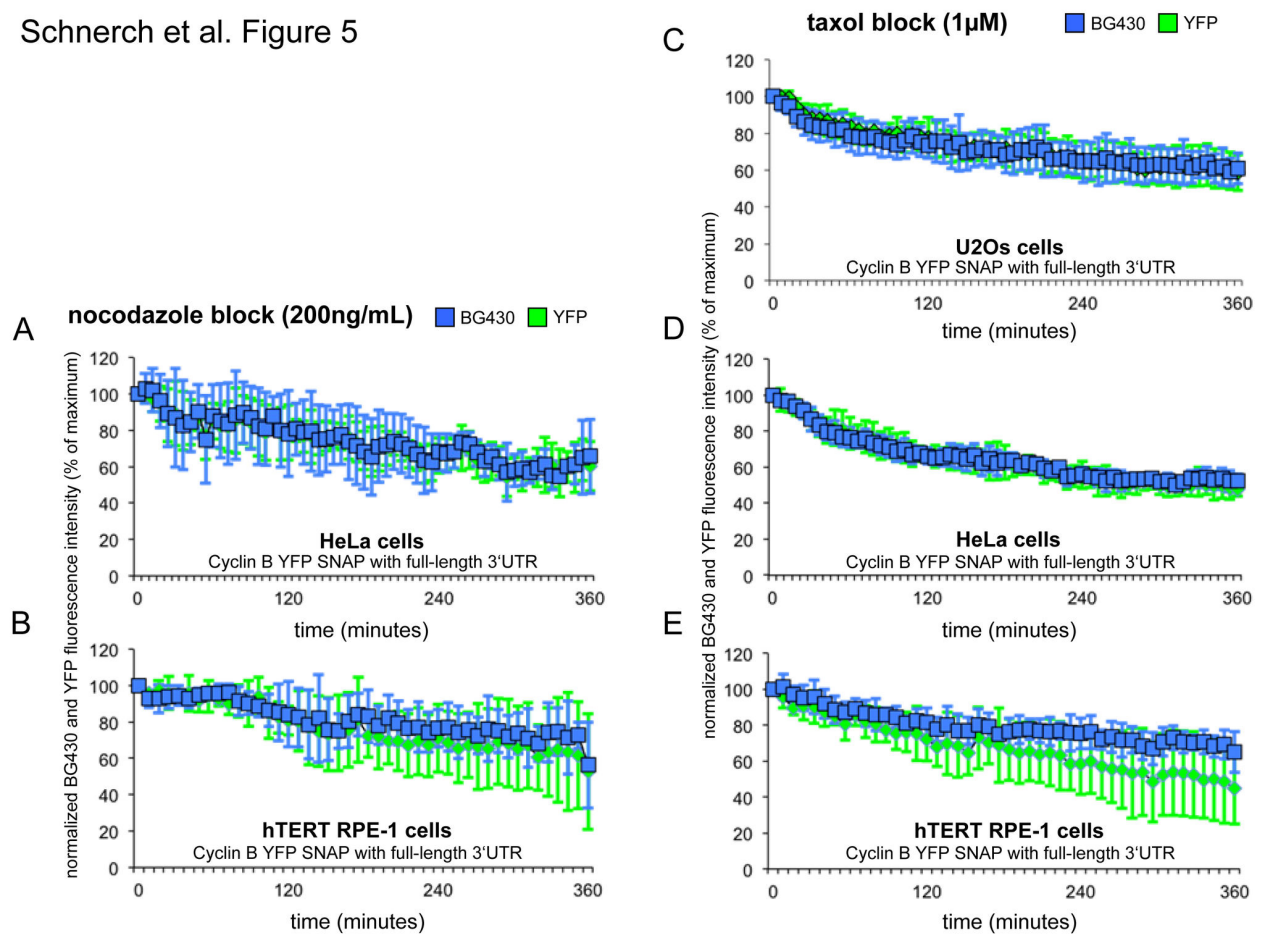
Characterization of cyclin B kinetics in the presence of the cyclin B 3’ UTR in human tumor- and hTERT-immortalized cells and in the presence of the microtubule-stabilizer taxol. YFP and BG430 fluorescence intensity curves as determined by live-cell imaging of U2Os, HeLa and hTERT RPE-1 cells expressing the CYS reporter with a long 3’ UTR sequence. HeLa and hTERT RPE-1 cells that are arrested in a mitotic block using a final nocodazole concentration of 200ng/mL in the growth medium are depicted in (A) and (B) (mean fluorescence intensities +/- SD are indicated, n=4). U2Os, HeLa and hTERT RPE-1 cells that are arrested in a mitotic block using a final taxol concentration in the growth medium of 1µM are depicted in (C, D and E) (mean fluorescence intensities +/- SD are indicated, n=4). For every shown experimental setting the analyzed cells are representatives of three independent experiments each of which included at least 50.000 cells.

To address the question of whether or not different cell lines of human origin behave in a way similar to U2Os cells, we generated HeLa (cervical cancer cell line) and hTERT RPE-1 cells (hTERT-immortalized retinal pigment epithelial cell line) which stably expressed the CYS reporter protein from an expression cassette also encoding the long cyclin B 3’ UTR sequence. During a mitotic block induced by nocodazole in HeLa- and hTERT RPE-1 cells, we noted a decline in YFP- and BG430-fluorescence intensities (Figure 5 A, B) which was very similar to that seen in U2Os cells (Figure 4F).

### Insufficiency of the human endogenous cyclin B 3’ UTR to contribute to synthesis of cyclin B in a mitotic block is also observed in taxol-arrested cells

A mitotic block can be induced by either disrupting the mitotic spindle by microtubule-depolymerizing agents such as nocodazole, or by exposure of cells to microtubule-stabilizing agents such as taxol. We therefore addressed the question as to whether the human endogenous cyclin B 3’ UTR mediates active synthesis in a mitotic block induced by the microtubule-stabilizer taxol. To this end, U2Os, HeLa and hTERT RPE-1 cells expressing the CYS reporter protein from an expression cassette and also containing the long cyclin B 3’ UTR sequence were treated with taxol at a final concentration of 1µM. For all three investigated cell lines we noted a decline in YFP- and BG430-fluorescence intensities similar to those observed in nocodazole-treated cells (Figure 5 C, D, E).

## Discussion

We describe here a novel double-chimeric SNAP tag-based system that allowed us to simultaneously monitor whole protein expression, synthesis/accumulation and degradation of cyclin B at the single cell level. Since recent reports suggest that cyclin B is actively synthesized during mitosis [13-15], we engineered a sequence encoding a double-chimeric cyclin B reporter molecule to study synthesis/accumulation of cyclin B during a mitotic block by live-cell imaging. We provide evidence that this system allows the measurement of both whole cyclin B expression and cyclin B degradation throughout most of the cell cycle. Measurements were restricted when the cell underwent morphological changes, such as during mitotic entry and exit, since quantification of fluorescence intensity by widefield microscopy led to notable increases in fluorescence intensity per area secondary to mitotic rounding. To accomplish this we defined a time window during mitosis which we named the isomorphic window, ranging from prometaphase to early anaphase, where the cell keeps a stable spheroidal shape [7]. Measuring fluorescence intensities at time points within the isomorphic window ensured that changes in fluorescence intensities were not caused by morphological changes. In addition, we did not detect any significant or abrupt changes in the morphology of cells in the G1 and S/G2 phases, as evidenced by the corresponding picture galleries, which allowed the quantification of fluorescence intensity in interphase cells as well. By quantifying whole protein expression and degradation at the single cell level, we were able to calculate the extent to which cyclin B was actively synthesized and accumulated during the S- and G2-phase, providing a proof of principle for the functionality of our system. Moreover, since both BG430 and YFP fluorescence intensity measurements were derived from the same object, the synthesis rate, as defined by the difference in YFP and BG430 fluorescence intensities, becomes independent of changes in cell shape.

The Scan^R-based algorithm allowed us to follow large numbers of cells at the same time, facilitating the study of single cells displaying spontaneous chromosomal misalignments. This condition is very rare in unperturbed cell populations [7] and is associated with an active SAC. To investigate cyclin B turnover during a stable mitotic block, we challenged U2Os, HeLa and hTERT RPE-1 cells with the microtubule-disrupting agent nocodazole and the microtubule-stabilizing agent taxol to induce sustained activation of the SAC. We did not detect any active synthesis/accumulation of cyclin B during mitotic blockage induced by spontaneous chromosomal misalignment in U2Os cells, or poisoning of the mitotic spindle by either nocodazole or taxol in U2Os, HeLa or hTERT RPE-1 cells, regardless of whether the 3’ UTR was present or not.

Recent studies have demonstrated that interference with mitotic exit is a better therapeutic rationale for cancer than activation of the SAC [24,25]. Mitotic exit can be more efficiently blocked by direct inhibition of APC/C-dependent proteolysis, while mitotic slippage may occur after SAC activation following spindle poison treatment [24-26]. Furthermore, an increase in expression levels of the APC/C substrate protein cyclin B can boost a mitotic block in spindle poison treated cells by attenuating mitotic slippage [10,18,19]. Since synthesis of cyclin B takes place in mitosis in murine and human cells [14,15] we assumed that conserved networks control the strength of a mitotic block. In mice cyclin B synthesis was shown to be enhanced by the presence of the endogenous cyclin B 3’ UTR sequence in Cdc20 hypomorphic cells [14]. This study was conducted using a cyclin B reporter that was tagged in a similar way with Venus, indicating that C-terminal tagging of cyclin B does not block 3'UTR-dependent translation [14]. Therefore, the objective of our study was to test the role of the human cyclin B 3’ UTR in cyclin B synthesis during a mitotic block in human cells. Our data indicate that in human tumor cell lines (U2Os and HeLa) and immortalized human epithelial cells (hTERT RPE-1) the endogenous cyclin B 3’ UTR is not sufficient to enhance the synthesis of cyclin B during a mitotic block. We found that this finding holds true for depolymerizing (nocodazole) as well as stabilizing spindle poisons (taxol). However, there are several differences between our study and the previous study in mice, which make a direct comparison difficult. The most prominent difference is that 3’ UTR-dependent accumulation of cyclin B in mitosis was primarily observed in mouse embryonic fibroblasts (MEFs) with a Cdc20 hypomorphic background [14].

In conclusion, we provide evidence that the human cyclin B 3'UTR, fused to the coding sequence of our cyclin B reporter, is not capable of inducing cyclin B synthesis during a mitotic block in human cells per se. The reporter system described here provides a valuable tool to gain further insights into the regulation of cyclin B synthesis during mitosis.

## Supporting Information

Figure S1 **Degradation kinetics of our CYS reporter molecule in the absence and presence of the full-length human endogenous cyclin B 3’ UTR.** Cell cycle kinetics and CYS expression levels of U2Os cells expressing the CYS expression cassettes with and without the human endogenous 3’UTR are shown. Cell cycle distribution at the indicated time points during the nocodazole release was assessed by propidium iodide staining (A, B). Expression levels of wild-type cyclin B and of our CYS reporter protein were assessed by Western Blot (C). In analogy to the experimental setting used for live-cell imaging, the lowest cyclin B/CYS expression levels in G1 phase were subtracted and expression levels were normalized based on maximum expression during the time of release from the nocodazole block (D).
(TIF)

Movie S1 **Degradation and synthesis of the double-chimeric cyclin B reporter throughout the cell cycle by live-cell imaging at the single-cell level in U2Os cells.** The movie depicts two single cells that express the CYS reporter molecule and were stained with BG430. An overlay of YFP (cyclin B expression), BG430 (cyclin B degradation) and mCherry (chromosomes) fluorescence is presented. Both cells exhibit a rapid decline in YFP and BG430 fluorescence intensity (turquoise fluorescence) during mitotic exit. Over time, all daughter cells show a steady increase in YFP fluorescence intensity (green fluorescence) indicating re-accumulation of the CYS reporter molecule.
(AVI)
