# Supplementary figures and images for: The 3' Untranslated Region of the Cyclin B mRNA Is Not Sufficient to Enhance the Synthesis of Cyclin B during a Mitotic Block in Human Cells
Source: PLoS One. 2013 Sep 13;8(9):e74379. doi: 10.1371/journal.pone.0074379 (PMC3772928; doi:10.1371/journal.pone.0074379)

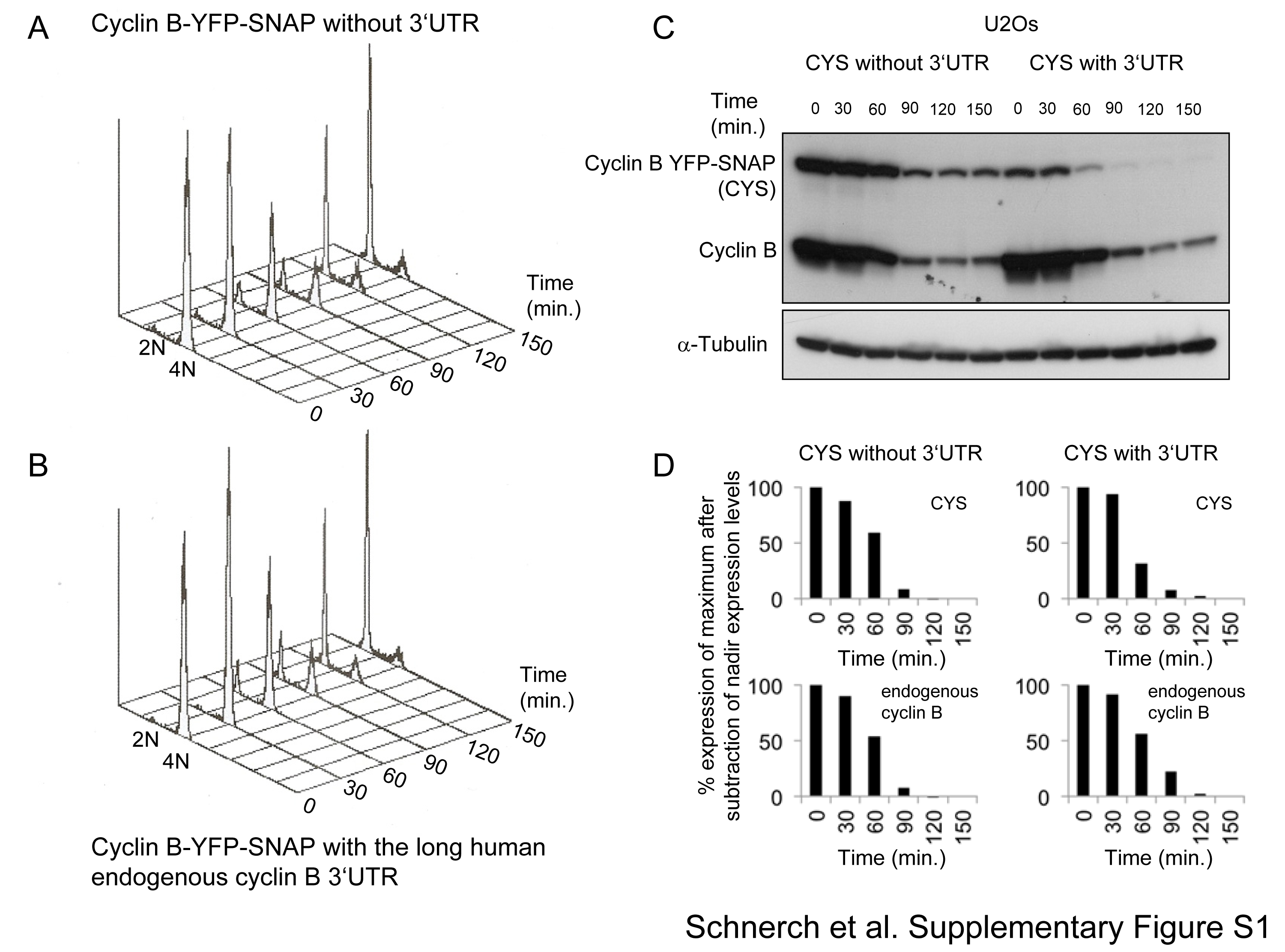

Supplement: Figure S1 — Degradation kinetics of our CYS reporter molecule in the absence and presence of the full-length human endogenous cyclin B 3’ UTR. Cell cycle kinetics and CYS expression levels of U2Os cells expressing the CYS expression cassettes with and without the human endogenous 3’UTR are shown. Cell cycle distribution at the indicated time points during the nocodazole release was assessed by propidium iodide staining (A, B). Expression levels of wild-type cyclin B and of our CYS reporter protein were assessed by Western Blot (C). In analogy to the experimental setting used for live-cell imaging, the lowest cyclin B/CYS expression levels in G1 phase were subtracted and expression levels were normalized based on maximum expression during the time of release from the nocodazole block (D). (TIF) [file pone.0074379.s001.tif]
